# Harnessing Edible Wild Fruits: Sustainability and Health Aspects

**DOI:** 10.3390/nu17030412

**Published:** 2025-01-23

**Authors:** Lorena González-Zamorano, Rosa M. Cámara, Patricia Morales, Montaña Cámara

**Affiliations:** Nutrition and Food Science Department, Pharmacy Faculty, Complutense University of Madrid, 28040 Madrid, Spain; lorego06@ucm.es (L.G.-Z.); rosacama@ucm.es (R.M.C.); patmoral@ucm.es (P.M.)

**Keywords:** plant foods, human health, sustainability treaties, food regulation, wild resources

## Abstract

Our health, well-being, and development are intrinsically linked to the preservation of biodiversity. This situation has driven the establishment of numerous treaties, international agreements, and regulatory frameworks that address sustainable food systems from multiple perspectives, including agriculture, food security, biodiversity, and environmental sustainability. The objective of this study is to review the potential of wild edible fruits in terms of sustainability and implications for human health. Specifically, this work examines the contribution of these fruits to promoting biodiversity, and their support for sustainable food systems as well as their beneficial role in human health. Additionally, it considers the evolution of relevant international treaties related to the preservation of wild fruits. An in-depth review of international treaties related to the conservation of wild fruits was conducted by consulting information available on official websites of international organizations such as the United Nations and the Food and Agriculture Organization of the United Nations (FAO), among others. Next, a review of the sustainability and health benefits of edible wild fruits was performed. Results showed that although numerous studies have demonstrated the health benefits of wild edible fruits, there is still a lack of scientific evidence showing that the use of these species could have positive effects not only on human health and well-being but also on the environment and biodiversity. Thus, integrating these fruits into sustainable practices could play a key role in supporting future food security and the well-being of communities.

## 1. Introduction

Biodiversity and current food systems are closely interrelated. Conserving biodiversity is crucial for maintaining the health of ecosystems and the services they provide. At the same time, our health, well-being, and development are intrinsically linked to the preservation of this biodiversity [1]. Over the years, the scientific community has increasingly recognized the threats that these issues pose to our planet. Concerns about global warming and biodiversity loss arose in the late 20th century [2], as humanity began to realize the global-scale damage it was inflicting on nature, with potentially unpredictable consequences.

This situation has driven the establishment of numerous treaties, international agreements, and regulatory frameworks that address sustainable food systems from multiple perspectives, including agriculture, food security, biodiversity, and environmental sustainability. These measures aim to promote responsible management and use of natural resources, ensuring that food production processes are not only efficient and productive but also environmentally friendly and sustainable in the long term.

In terms of food supply, as the nutritional requirements of the population continue to increase exponentially, climate change increasingly hinders food cultivation [3]. Factors such as rising atmospheric CO_2_ levels, declining pollination services, and soil degradation directly or indirectly impact various stages of our food system, from production and processing to consumption and waste generation [4]. Simultaneously, the lack of access to safe, nutritious, and sufficient food in both quantity and quality leads to malnutrition, disease, and overall health deterioration [5].

The drastic rise in the global population, now estimated to be three times larger than in the mid-20th century and projected to continue growing exponentially [6], will demand the production of safe, nutritious, and sustainable food. This will require effective resource management to meet the needs of an expanding population [7].

Considering the immense value that biodiversity brings to economic and social development, and recognizing that the global food system relies on a limited number of plant species, some authors highlight the importance of revaluing numerous nutrient-rich varieties that were historically used [8]. Previous studies noted that improving the knowledge on natural resources offered by biodiversity, such as edible wild plant species, could help us understand not only their agro-industrial potential but also their potential nutritional value [9]. Wild plant species could be an interesting source of bioactive compounds with antioxidant and biological activity [10], which are associated with health benefits, including the prevention of cardiovascular diseases, neurological disorders, diabetes, and cancer [11]. The objective of this study is to review the potential of wild edible fruits in terms of sustainability and implications for human health. Specifically, given the well-established link between biodiversity and sustainability, as well as the relationship between the use of wild plants and human health, this work examines the contribution of these fruits to promoting a relation between biodiversity and their support for sustainable food systems, as well as their beneficial role in human health, by demonstrating that the employment and conservation of wild edible fruits can simultaneously enhance ecological diversity, improve human nutrition, and support resilient food production. On one hand, the support provided by wild edible fruits to sustainable food systems will be evaluated, considering economic, ecological, social, and cultural factors. On the other hand, this study will explore the beneficial effects of wild edible fruits on human health, highlighting their nutritional value, their use in traditional medicine, and their health benefits supported by scientific evidence. In this way, the aim of this study is to establish a connection between the resources that biodiversity can offer us, especially wild edible fruits, and human health.

Additionally, it considers the evolution of relevant international treaties related to the preservation of wild fruits, analyzing their effectiveness in conserving these resources and identifying potential areas for improvement.

## 2. Materials and Methods

To comprehensively examine the interplay between wild edible fruits, biodiversity conservation, and human health, this study employed a multi-pronged approach.

First, an in-depth review of international treaties related to the conservation of wild fruits was conducted by consulting information available on official websites of international organizations such as the United Nations, the Food and Agriculture Organization of the United Nations (FAO), the Convention on Biological Diversity (CBD), and the European Green Deal, among others. Official pages and selected reports detailing the goals achieved during specific time periods were also reviewed.

These treaties were organized chronologically (by year of establishment), with a focus on those most relevant to biodiversity that may involve wild species as a food sources, while excluding treaties exclusively related to water protection or the use of persistent organic pollutants (POPs).

Next, a review of the sustainability and health benefits of edible wild fruits was performed. This included a PubMed search from 2000 to the present, using the keywords “sustainability”, “wild edible fruits” and “human health”. Studies discussing mushrooms were excluded. The focus was on articles that explored the economic, ecological, social, or cultural aspects of edible wild fruits and that provided evidence of their traditional medicinal uses or their phytochemical composition.

Furthermore, the references cited in the retrieved articles were reviewed to identify additional relevant studies and expand the scope of the literature review. Additionally, to further expand the knowledge base related to the study objectives, articles from databases such as Web of Science and Google Scholar were also consulted.

## 3. International Treaties Addressing Sustainable Plant Foods

**Figure 1:**
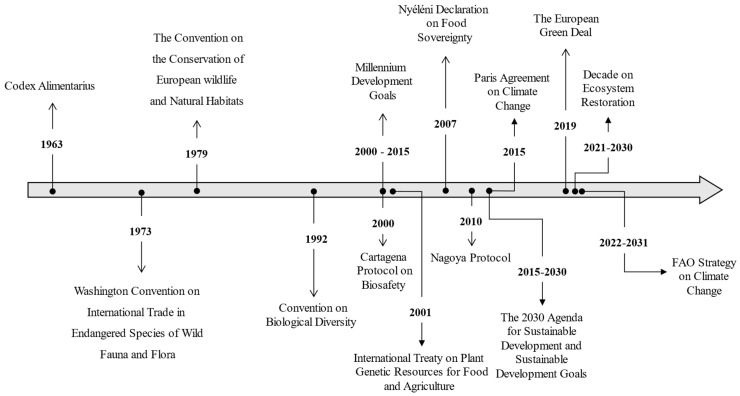
Evolution in the emergence of international and European treaties or strategies addressing sustainable food systems.

A range of international treaties and agreements have been established to address the urgent need for more sustainable food systems (Figure 1). These initiatives respond to the growing global demand for sustainable agricultural practices, the conservation of biodiversity, and the mitigation of climate change impacts on food security.

### 3.1. Codex Alimentarius

The Codex Alimentarius Commission was established in 1963 during the World Health Conference organized by the Food and Agriculture Organization of the United Nations (FAO) and the World Health Organization (WHO). It created a comprehensive collection of internationally harmonized food standards, guidelines, and codes of practice, known as the Codex Alimentarius. Since its inception, the Codex has developed internationally accepted food standards to protect consumer health and ensure fair trade practices in the global food market [12].

Since its creation, the Codex Alimentarius has evolved in response to emerging public health needs. In recent years, with the rise of sustainability policies, the Codex has sought to integrate sustainability considerations into its guidelines. Today, the Codex is working to support the achievement of the Sustainable Development Goals (SDGs) by strengthening regulations aimed at providing safer and higher-quality food for all. It currently addresses six of the seventeen SDGs. The SDG most closely linked to food insecurity is Goal 2 (Zero Hunger), where Codex standards address trade and emphasize the need to correct and prevent trade restrictions [13].

Despite these efforts to incorporate sustainability, the Codex Alimentarius does not yet have specific regulations related to wild plants. However, given the growing interest in wild foods and the FAO’s ongoing work on biodiversity conservation and sustainable wild plant harvesting practices, there may be future developments of norms concerning these foods.

### 3.2. Washington Convention (CITES)

CITES (Convention on International Trade in Endangered Species of Wild Fauna and Flora) is an international intergovernmental agreement that entered into force in 1975. It aims to ensure international transboundary movement of endangered specimens of plants and wild animals, avoiding unsustainable exploitation through international trade and trafficking. The protection extends to animals and plants, whether alive or dead, and their parts, derivatives, or products containing them. Countries participating designate one or more Management Authorities to regulate the authorization and certificate necessary for each species listed in Annexes A and B, and the Annexes of EU Regulation 338/97. Scientific committees advise on the effects of trafficking and trade on the species and establish the points of introduction authorized by each country. This treaty has the aim of ensuring that international trade of wild animals and plants is legal, sustainable, and traceable.

### 3.3. Convention on the Conservation of European Wildlife and Natural Habitats

This convention was the first international treaty that considers comprehensive land management to be necessary for the conservation of both fauna and flora. The Convention on the Conservation of European Wildlife and Natural Habitats was signed in Bern in 1979 and made mandatory in 1982 initially among 19 member states (currently 51 contracting parties, including four African states and the European Union). The depositary was the Council of Europe, and expert groups set up by its Standing Committee planned conservation strategies. One of the major advances of this convention was the incorporation of the conservancy policy in the economic planification of land use for the preservation of habitats (Art. 3 and 4) and the consideration of a global approach for the conservation of wildlife, with a necessary international cooperation. International conservation actions were also established to protect endangered migratory species that were also considered in the Bonn Convention for the conservancy of migratory species.

With an emerging environmental awareness, the Bern Convention was the first international treaty that established specific Action Plans for the conservation of plants and animals, considering strictly protected species (Annex II) and those requiring special management measures (Annex III). In this convention was also considered, for the first time, the importance of invasive alien species for the preservation of biodiversity. As a consequence of this regulation, national catalogues of protected species were created in the member states. The Bern Convention has recently adopted a Strategic Plan to 2030 on nature conservation with the vision of “healthy nature for healthy people” to improve the lives of people and contribute to the health of the planet.

### 3.4. Convention on Biological Diversity

In response to concerns about biodiversity loss and climate change, the United Nations Environment Programme (UNEP) convened a group of experts in November 1988 within the Ad Hoc Working Group to explore the possibility of establishing a new international treaty on biological diversity. This initiative culminated in the creation of a legal instrument that was adopted on 22 May 1992 at the Nairobi Conference, and opened for signature on 5 June 1992 [14].

The Convention on Biological Diversity (CBD) aims to conserve biological diversity, promote the sustainable use of its components, and ensure the fair and equitable sharing of benefits arising from genetic resources. The CBD represents a global commitment to addressing the challenges related to biodiversity and emphasizes the importance of preserving the natural world for current and future generations. The Convention officially came into force on 29 December 1993, establishing itself as a nearly universal international treaty with over 196 parties.

Since 1993, the CDB has been recognized as a key framework for addressing global biodiversity challenges, including the sustainable use of biological resources. It advocates for the responsible use of these resources to prevent depletion and environmental degradation, ensuring that future generations can continue to benefit from biodiversity.

Wild species, including wild edible fruits, are a critical component of global biodiversity, and the CDB has played a key role in promoting their sustainable management, ensuring that resources are harvested in ways that do not lead to depletion or environmental harm. In this context, in 2010, the CBD adopted the Strategic Plan for Biodiversity 2011–2020 and the Aichi Biodiversity Targets, which included references to wild species, both related species and those of socio-economic and cultural value [15]. These targets emphasized the sustainable management of agricultural systems and proposed maintaining the conservation status of species, as well as the restoration of ecosystems. The Convention on Biological Diversity, EU Patent Directive, and the Agreement on Trade-related Aspects of Intellectual Property Rights (TRIPS) of the World Trade Organization (WTO) are influencing each other; when one strengthens its measures, the other adopts tougher measures that can facilitate technological innovation, improve conservation, or enhance international equity, sometimes coming into conflict [16]. It is necessary to highlight the complexity of commercializing biodiversity via patents of wild plants that really ensure equitable benefit. This may involve sharing, associated with the commercialization of the patent, as different wild plants’ case studies demonstrated [17].

As a consequence of the increment in the use of biotechnology in agricultural production, the Cartagena Protocol on Biosafety was established in the year 2000, as a supplementary international agreement to the CDB. This protocol was specifically focused on the use of living modified organisms from modern biotechnology in agriculture production to prevent their possible adverse effects on biological diversity.

### 3.5. The Millennium Development Goals (MDGs)

In 2000, the United Nations General Assembly established a set of eight international objectives aimed at addressing some of the most pressing global challenges, particularly in the areas of poverty reduction, health, education, gender equality, and environmental sustainability, which were to be pursued until 2015 [18]. Although these MDGs did not specifically address the use of wild plants, such species can be indirectly related to the goals outlined in the framework.

MDG 7, which focused on ensuring environmental sustainability, is closely linked to the sustainable use of wild resources, as it emphasized the need for national policies and programs that promote environmental conservation. Similarly, MDG 1, which aimed to eradicate extreme poverty and hunger, is also relevant to wild plants, as they play a crucial role in food security. Sustainable use of wild edible plants could contribute to the conservation of these species, which, in turn, could enhance future food security.

In 2015, as the period for the MDGs came to an end, the United Nations issued a report assessing progress towards these goals. The report indicated that while significant progress had been made in reducing hunger, millions of people continue to live in poverty and hunger [19]. Recognizing the potential of wild plant species, both in terms of nutrition and health, could foster their sustainable use and, consequently, help achieve current goals related to poverty and hunger.

The report also referenced the International Union for Conservation of Nature (IUCN) Red List Index, a list that highlights the increasing extinction risk faced by various species and the urgent need for guidelines aimed at preventing biodiversity loss [19]. The IUCN Red List Index for Plants takes into account global conservation assessments for only one third (31%) of known wild food plants [20]. The MDGs included the goal to “significantly reduce the rate of biodiversity loss by 2010” through the United Nations Environment Programme World Conservation Monitoring Centre (UNEP-WCMC), which, together with IUCN support, monitored the progress and compliance of the MDGs.

In this context, such regulatory guidelines could promote the sustainable use of wild plant species as part of conservation strategies. Similarly, this approach would not only contribute to the preservation of biodiversity but also improve human health and well-being. This new approach recognizes that poverty reduction and environmental management must be considered together.

### 3.6. International Treaty on Plant Genetic Resources for Food and Agriculture (ITPGRFA)

After years of intense negotiations led by the Commission on Genetic Resources for Food and Agriculture, under the auspices of the Food and Agriculture Organization of the United Nations (FAO), a total of 180 countries adopted the International Treaty on Plant Genetic Resources for Food and Agriculture on 3 November 2001. Four years later, in 2004, the Treaty entered into force and aims to ensure the conservation and sustainable use of plant genetic resources, while also guaranteeing the fair and equitable sharing of benefits derived from their use. This aligns with the objectives of the Convention on Biological Diversity, supporting sustainable agriculture and food security [21]. The Treaty upholds national sovereignty over genetic resources, emphasizing that countries must ensure access to these resources in accordance with their national legislation, while respecting the rights of indigenous peoples and local communities.

Although the Treaty primarily focuses on agricultural crops, it also plays an important role in the conservation and sustainable use of wild plant resources [22], particularly through its provisions under Article 5. This article promotes the collection of plant genetic resources for food, alongside the traditional knowledge associated with them. Furthermore, the Treaty advocates for the in situ conservation of wild relatives of crops and wild plants used for food production, including in protected areas, with support for the efforts of indigenous and local communities. The first FAO State of the World’s Plant Genetic Resources report [23] laid the groundwork for the development of the first Global Action Plan for the Conservation and Sustainable Use of Plant Genetic Resources for Food and Agriculture (PGRFA), which was approved in 1996. This plan aimed to highlight the importance of these resources and to promote, as stated in the International Treaty on Plant Genetic Resources for Food and Agriculture, the conservation of genetic diversity, particularly in situ [24]. Furthermore, the second FAO State of the World’s Plant Genetic Resources report emphasized the global neglect of wild food plants, especially those outside protected areas. Based on this report, the Second Global Plan of Action for Plant Genetic Resources for Food and Agriculture was developed by the Commission on Genetic Resources for Food and Agriculture and the FAO in 2011. This plan reiterated the value of wild resources for both the economy and food security [25,26].

Additionally, Article 6 of the Treaty highlights the promotion of the expanded use of underutilized crop varieties and species [22], which includes wild plant species that could contribute to enhancing the genetic diversity of cultivated crops. Therefore, integrating these wild resources into sustainable agricultural practices, in accordance with the guidelines established by international treaties, could play a key role in strengthening global food systems.

### 3.7. Nyéléni Declaration on Food Sovereignty

On 27 February 2007, following a meeting of over 500 representatives from 80 countries convened by the International Forum on Food Sovereignty in Nyéléni, Mali, a document was drafted outlining fundamental principles and objectives for achieving food sovereignty. The declaration defined “the right of peoples to nutritious and culturally appropriate food produced through ecologically sound and sustainable methods, and their right to define their own food and agriculture systems… It defends the interests and inclusion of the next generation” [27].

The Nyéléni Declaration is recognized as a foundational document that guides various communities and organizations in their efforts to promote sustainable food systems. It represents a milestone in the food sovereignty movement, encompassing aspects such as local control of food systems, sustainability, ecological agricultural practices, and the protection of traditional knowledge. Additionally, the Nyéléni Declaration holds particular significance for rural communities, indigenous peoples, and small-scale farmers, as it emphasizes the importance of local economies and markets while affirming the right of these populations to access and manage the land and biodiversity that sustain food production. By implementing these principles in rural communities, it could play a crucial role in preserving wild edible species and safeguarding the traditional knowledge associated with them. The Declaration underscores the need for the conservation and rehabilitation of rural environments, landscapes, and food traditions, promoting the sustainable management of biodiversity as a fundamental aspect of food sovereignty. At the same time, the conservation and promotion of knowledge regarding wild species would have an impact on food security and contribute to the development of local markets, thereby strengthening the local economy.

### 3.8. Nagoya Protocol

Although access to biological resources has historically been regulated by states through their own laws, access to genetic resources and the traditional knowledge derived from them has often been controlled by a very limited number of states [28]. All of this led to tension and imbalance between different regions of the world. Developing countries, abundant in biodiversity, had limited capacity to convert genetic resources and the traditional knowledge associated with them into industrial products. Meanwhile, developed countries, despite their advanced technological capabilities for transforming genetic resources, lacked the essential resources and raw materials [29]. This highlighted the need for a legislative measure to address the imbalance between different regions of the world in terms of biodiversity. The Nagoya Protocol was adopted on 29 October 2010, in Nagoya, Japan, following the tenth meeting of the Conference of the Parties (COP) to the Convention on Biological Diversity (CBD), and the decision made in the Strategic Plan for Biodiversity 2011–2020 and the Aichi Biodiversity Targets. The Protocol was adopted to fulfil one of the objectives of the Convention on Biological Diversity: Access to Genetic Resources and Fair and Equitable Sharing of Benefits Arising from Their Utilization (ABS). During this meeting, in line with the strategic objective of enhancing the benefits of biodiversity and ecosystem services for all, Target 16 proposed that the Nagoya Protocol should enter into force before 2015. Finally, in line with these objectives, the Nagoya Protocol entered into force on 12 October 2014 [30].

The Nagoya Protocol aims to ensure that all states commit to establishing legislative measures that guarantee genetic resources used within their jurisdiction have been accessed in accordance with the national framework of the provider country. This will result in greater transparency in sectors that utilize genetic resources and a more equitable transfer of benefits to the provider countries, thereby contributing to the conservation of biological diversity and the sustainable use of its components.

Moreover, implementing the Nagoya Protocol would help respect, preserve, and maintain the knowledge, innovations, and practices of indigenous and local communities. It would also recognize these communities’ contributions to scientific and technological advancements derived from traditional knowledge.

### 3.9. Paris Agreement on Climate Change

The Paris Agreement is a legally binding international treaty on climate change, which came into force on 4 November 2016, following its adoption by 196 parties at COP21 in Paris in 2015. In addition to addressing the challenges posed by climate change, the Agreement underscores the importance of adaptation in the agricultural sector and promotes sustainable agricultural practices. These practices are essential not only for reducing emissions but also for enhancing resilience to climate impacts, ultimately contributing to improved food security, where wild edible plants are essential for developing crops better adapted to climate stressors [31].

From the perspective of biodiversity conservation and climate change adaptation, wild fruits have the potential to play a significant role in fostering more resilient agricultural systems. By supporting biodiversity and mitigating the effects of climate change, wild edible plants present an opportunity to strengthen ecosystem resilience. In terms of food security, the use of edible wild fruits could be critical for diversifying food sources. These wild resources, capable of thriving in extreme conditions, may become an important source of nutrients in regions vulnerable to climate change, offering a more sustainable alternative to conventional crops. The traits of wild edible plants make them particularly valuable for developing crops that are more resilient to climate stressors.

Furthermore, the Paris Agreement recognizes the importance of traditional knowledge in the conservation and promotion of biodiversity, particularly the knowledge of local and indigenous communities, contributing to cultural sustainability.

### 3.10. 2030 Agenda for Sustainable Development

In 2015, the United Nations adopted the 2030 Agenda for Sustainable Development [32], aiming to implement specific strategies to eradicate poverty, promote economic growth, and address various social needs, including education, healthcare, and environmental protection. The agenda consists of 17 Sustainable Development Goals (SDGs) and 169 Targets that serve as a universal call to action for countries to end poverty, protect the planet, and ensure prosperity for all. The 2030 Agenda seeks to balance the three dimensions of sustainable development, economic, social, and environmental, by providing a comprehensive framework for global development and cooperation.

The role of wild species within the agenda is reflected in SDG 2 (Zero Hunger), aligning with the objectives of the CBD and the International Treaty on Plant Genetic Resources for Food and Agriculture. Additionally, it is related to SDG 15 (Life on Land) due to its promotion of biodiversity conservation and the fair and equitable access and sharing of benefits derived from the use of genetic resources, recognizing the value of wild plants for their economic and social benefits necessary for sustainable development [15].

The European Green Deal, launched in December 2019, is a growth strategy from the European Union aimed at facilitating a green transition with the ultimate goal of achieving climate neutrality by 2050 [33]. This treaty emerged as a European framework to support the objectives outlined in the Paris Agreement on Climate Change, underscoring the need for all policy areas to contribute to the fight against climate change.

In relation to food systems, one of the key objectives of the European Green Deal for 2050 is to mobilize the industry towards a clean and circular economy, recognizing that over 90% of biodiversity loss and water stress can be attributed to resource extraction and the transformation of materials, fuels, and food.

Another critical aspect of the European Green Deal linked to food is the proposal for a fair, healthy, and environmentally sustainable food system, which led to the development of the Farm to Fork Strategy. This strategy, adopted on 20 October 2021, aims to ensure that European food is recognized not only for its safety, quality, and nutritional value, but also as sustainable food [34]. To achieve this, the strategy includes proposals to improve the entire food value chain and to raise awareness among citizens about sustainable consumption.

Within these proposals, measures were introduced to identify innovative food products, where wild edible fruits could play a significant role. These resources are currently underutilized but are well known for their nutritional qualities. Moreover, the sustainable use of wild resources could contribute to another key goal of the European Green Deal: the preservation and restoration of ecosystems and biodiversity.

### 3.11. UN Decade on Ecosystem Restoration

The United Nations Decade on Ecosystem Restoration, established by the UN General Assembly in 2021 and running until 2030, aims to halt ecosystem degradation and restore ecosystems to meet global biodiversity protection goals for the benefit of both nature and humanity [35]. This resolution aligns with other European Union frameworks, such as the Sustainable Development Goals (SDGs), by emphasizing the importance of ecosystem restoration as a key strategy for achieving global sustainable development.

The resolution calls for the active participation of governments, international and regional organizations, and civil society in supporting the Decade’s implementation, working together towards sustainable global development.

In this context, raising public awareness about the nutritional value of wild edible fruits could play a significant role in supporting ecosystem protection initiatives. This awareness would contribute to efforts aimed at safeguarding biodiversity and fostering sustainable development practices.

### 3.12. FAO Strategy on Climate Change

The new FAO Strategy on Climate Change for the next ten years echoes the recognition of the Paris Agreement of the fundamental priority of safeguarding food security and ending hunger [36]. The principles guiding the Strategy are a focus on improving the living standards of all, in an economically, socially, and environmentally sustainable manner, And trying to involve all food value chain workers through Action Plans including rural, peri-urban, and urban areas. The strategy adopts a “no-one-size-fits-all” approach, as FAO’s climate action considers national circumstances and the diversity of contexts and capabilities across regions and countries and at the local level in terms of environmental, economic, and social development.

According to the principles of this strategy, the inclusion of wild edible plant species in the food value chain could significantly enhance food security by reducing reliance on crops that are more vulnerable to climate change. The cultivation of these species would not only promote climate-resilient agricultural practices and enhance biodiversity, but also foster more sustainable local markets, contributing to the development of the local economy. In this regard, wild edible plants could play a crucial role in improving both the sustainability and resilience of food systems in the face of changing climatic conditions.

## 4. Harnessing Edible Wild Fruits: Evolution of Relevant International Treaties Related to the Preservation of Wild Fruits

The impact of international treaties affecting wild edible fruits was very positive at the beginning and clearly attributable to the treaties established up to 1992 that set up the broad guidelines for biodiversity conservation, trade, and sustainable management. At present, the complexity of existing regulations in each country and the diversity of regulatory bodies involved makes it difficult to attribute success or failure in wild fruit preservation to a single treaty.

According to the latest FAO report on the state of the world’s biodiversity for food and agriculture (BFA), biodiversity is decreasing worldwide, and it remarked on the main threats: the loss and deterioration of agricultural and forest land due to climate change and high environmental impact agricultural practices, among others [37].

Future challenges that need to be addressed include the promotion of more efficient and resilient agriculture through the application of technology for hazard forecasting and monitoring, and greater government investment in conservation monitoring programs for recording, storing, and analyzing data on changes in the status of species and habitats. The recovery of less nutrient-demanding but more resilient wild varieties can be considered as part of these alternative agricultural strategies.

## 5. Scientific Literature Review on Sustainability and Health Aspects of Wild Edible Fruits

A search in PubMed with the inclusion criteria (keywords) “human health” and “sustainability” and restricted to the year 2000 up to today yielded 127,912 references, emphasizing the strong connection between environmental quality and human well-being. Additionally, using the keywords “wild edible fruits” and “human health” returned 119 references, highlighting the potential health benefits of consuming wild fruits, as noted by numerous authors. A further search combining “sustainability” and “wild edible fruits” produced 91 results, suggesting that the sustainable use of these natural resources plays a critical role in ecosystem conservation and food security. Finally, a search for the combination of “sustainability”, “wild edible fruits”, and “human health” resulted in 18 references (Table 1).

It became evident that since the adoption of international treaties focused on biodiversity conservation (1992), there has been exponential growth in the number of relevant publications. In fact, approximately 95% of the total references were published after 2000, highlighting the growing importance of sustainable policies aimed at combating biodiversity loss, ensuring food sovereignty, and addressing food insecurity. A review of the published articles provides valuable insights into the current state of scientific research on the links between wild edible fruits, sustainability, and human health. All the published articles highlighting the connection between wild fruits, sustainability, and human health emphasize the importance of conserving these natural resources to maintain ecological balance and promote human health, ensuring a sustainable future for the environment and, consequently, for the human population.

To organize all the reviewed information, Table 2 presents an overview of the sustainability aspects of various wild edible fruits, classifying them according to the three pillars of sustainability: environmental, social, and economic dimensions.

On the other hand, regarding the health aspects of wild edible fruits, it is worth mentioning that historically, wild edible species have served not only as food sources but also as forms of medicine in local populations since prehistoric times [48]. This knowledge remains particularly prevalent in rural areas, where traditional practices surrounding the use of wild edibles continue to thrive, as shown in Table 3. This table presents an overview of the health aspects of various wild edible fruits, as reported by the knowledge about the traditional uses of wild fruits in some regions. Pieroni et al. (2001) proposed the Cultural Significance of Wild Edibles (CFSI) index [49] to evaluate the potential of wild edible resources. The CFSI index includes the Food–Medicinal Role Index (FMRI), which categorizes wild edibles according to their health benefits and medicinal properties. The FMRI is divided into the following categories: ‘very high or medicinal food’ (value 5), ‘high or medicine for treating a specific disease’ (value 4), ‘moderately high or very healthy food’ (value 3), ‘moderately low, healthy food or unknown efficacy’ (value 2), and ‘unknown or possibly toxic’ (value 1). Additionally, Table 3 also highlights the health aspects of wild edible fruits based on scientific studies or analyses, which focus primarily on bioactivity and the content of bioactive compounds.

### 5.1. Importance of Edible Fruits as Sustainable Food Resource

Edible plant resources, including a wide range of species and plant parts such as fruits, vegetables, grains, legumes, nuts, spices, and herbs, have been essential to human survival and development for thousands of years. These plants have served as crucial sources of food and nutrition across all continents throughout history [52]. Over the past century, despite widespread recognition of the traditional knowledge held by various communities regarding the potential value of wild fruits, the industrialization and globalization of agriculture have significantly reshaped the lifestyles of these communities. Along with migratory movements and other factors, this has contributed to the gradual loss of traditional knowledge passed down through generations about the use of wild edible resources [48].

Consequently, while information on these resources at the national level remains scarce, an increasing body of research highlights the importance of wild edible species at the local level. As a result, numerous authors contend that the nutritional and economic potential of wild plants remains largely untapped and underutilized [20,53].

Wild edible plants constitute not only a genetic resource but also a vital part of local and indigenous food systems and cultural identities, with significant economic dimensions in many communities.

Wild edible fruits are among the most widely used non-timber forest products (NTFPs) that can significantly contribute to the objectives of the European Green Pact and the 2020 Biodiversity Strategy and help countries move towards a sustainable and circular bioeconomy. The economic importance of wild edible fruits was highlighted in the Rio international agreements and the European Ministerial Conference on the protection of forests in Europe, and there is growing evidence of the relevant role of wild edible fruits in contributing to the 17 SDGs. According to the FAO non-wood forest products white paper [54], the current and potential economic value of wild forest products is largely overlooked in official statistics and foresight analyses. There are few studies on the landscape ecology, economics, and conservation of WEFs, or the real economic impact of the consumption of edible wild fruits, as indicated by previous reviews of this aspect [55], but it is known that around 90% of European households regularly consume wild forest products, while 26% collect some type at least once a year for self-consumption or sale. According to FAO data [54], Europe is a central player in the international trade of wild forest products, including edible wild fruits, importing 4.2 billion euros, i.e., 50% of world imports, and exporting 3.4 billion euros, i.e., 40% of world exports. There is a lack of systematic and comparable studies, and better coordination among institutions is needed [54,55] to collect and file this information. Local producers and local associations have the information about the real impact in the food market, but data are not being systematically collected or are not available in many countries. Nevertheless, some specific studies have been carried out in tribal villages, with the study of 56 wild edible fruits in different parts of Orissa state in India [56] and Ethiopia [57].

Despite these local benefits, unsustainable harvesting practices and competition among harvesters, often driven by immediate economic incentives, exacerbate overexploitation [44]. While many harvesters acknowledge the decline in wild populations, the allure of immediate economic gains can outweigh long-term conservation incentives. Therefore, a synergistic approach combining robust legal protection measures with comprehensive education and awareness programs is essential to ensure the long-term sustainability of these valuable resources.

Furthermore, greater education on the use of wild foods could complement the food supply while also promoting sustainable land use [58].

The integration of wild fruit cultivation into complex mountain silvopastoral systems (SPs) makes use of underproductive land and provides shelter and food for fauna, in addition to offering complete vegetation cover that prevents erosion and contributes to the development of other plant communities. This type of traditional harvesting combines trees, pastures or crops, and/or livestock on the same plot of land, becoming an important element of cultural identity in many marginal and impoverished areas.

SPs have been revealed as a valuable farming system since their beginnings 7.5 million years ago [59,60], and they can be one of the best alternatives to restore degraded pastures, improve livestock productivity, and build resilience to climate change, as confirmed by the FAO through two action network experiences in Latin America [61].

The existence of international treaties for the protection of biodiversity and the rational use of natural resources had a positive impact, but there is still a need to reinforce or monitor the efficiency of current treatments and regulations that fail to reduce biodiversity loss. It is necessary to ensure compliance with biodiversity protection regulations and promote the sustainable development of indigenous communities, where these wild fruits constitute a fundamental part of their diet.

In Europe, the Habitats Directive was created to protect the habitats of tree, herbaceous, and shrub plant communities that could be at risk or under threat. Any land transformation action (crop planting, livestock, or wildlife management) must necessarily take into account conservation measures for species characteristic of these protected habitats. Additionally, other strategies have been developed in various countries under the umbrella of the aforementioned international treaties.

Furthermore, recent studies have highlighted the role of wild foods in climate change adaptation strategies [62], emphasizing the critical role of wild edible fruits in environmental sustainability. Global temperatures are rising, as shown in NOAA’s 2023 Annual Climate Report [63]. This increase in winter temperatures has led to earlier development in both cultivated and wild plant species, which have been studied in wine regions across different countries [64,65]. According to previous studies [66], wild edible plants have undergone changes in their variability, distribution, and flowering and ripening periods due to anthropic and environmental factors, showing a decrease in the abundance of wild plants and fungi that seriously affects the diet of indigenous communities around the world. Agricultural management practices must adapt, not only by adjusting growing seasons but also by incorporating new crop varieties as alternative crops to ensure stable food production [67]. On the other hand, the domestication of plants has significantly reduced genetic diversity in crops, resulting in the loss of important traits related to nutritional quality, environmental resilience, and disease resistance [68]. Currently, only about 150 plant species are widely cultivated globally, while there are approximately 30,000 edible species. The domestication of wild plants, based on traditional knowledge and genome editing, could offer a rapid method for developing new crops. Harnessing wild plant species, especially knowing the potential of wild fruits, represents a critical strategy for enhancing genetic diversity in modern crops, with the potential to improve food security sustainably [69]. This climate change adaptation of wild and semi-wild edible plants has been studied in Ethiopia [70]. These studies highlight the importance of adapting wild species to arid, unproductive soils, where they can withstand increased water stress and are less dependent on fertilizers.

Additionally, traditional knowledge, an integral part of the social dimension of sustainability, regarding wild edible fruits has been documented in tribal communities in arid zones in India [71], where the production and consumption of these fruits contribute to both dietary supplementation and economic activity in rural areas. For example, a study conducted in Ethiopia suggested that promoting strategic plans for the sustainable use and domestication of wild resources could contribute to socioeconomic improvement [72], while also supporting the achievement of several Sustainable Development Goals (SDGs).

All references for Table 2 emphasize the critical need to promote the sustainable utilization of wild fruits (which is included within the environmental benefits that wild edible fruits could provide for sustainability). Despite the observed decline in knowledge and interest in these species, wild plants continue to play a vital role in the diets of many rural communities. However, their availability has decreased in some regions due to a confluence of factors, including overexploitation, population growth, and climate change. In these areas, the implementation of robust conservation policies is crucial to mitigate the threats to wild edible plants [73]. Consequently, the establishment of international agreements that specifically address threats such as agricultural expansion, timber extraction, overexploitation, overgrazing, and the impact of invasive species is paramount. Concurrently, the dissemination of knowledge to local communities is essential to significantly reduce pressures on these species.

### 5.2. Benefits of Wild Edible Fruits in Health Promotion

Two main approaches have been found to demonstrate the relationship between wild edible fruits and health: traditional methods and experimental assays. In many instances, traditional knowledge aligns with scientific evidence. For example, *Choerospondias axillaris*, which has a value of 5 on the FMRI index, has been traditionally used in China for the treatment of cardiovascular diseases for many years [41] and is well known for its high levels of antioxidant compounds. Notably, fruit extracts from this plant have been shown to contain 568 mg of gallic acid equivalents/g dry material [74], which may contribute to its potential health benefits, including antiarrhythmic effects that have been demonstrated to persist even after in vitro simulated digestion [75,76].

Similarly, *Gardenia jasminoides* J.Ellis is a popular fruit in China, recognized as a “medicine-food”, and has been found to contain iridoid glycosides such as geniposide, which inhibit atherosclerosis by enhancing macrophage autophagy and lipid transport [77]. This fruit also scores 5 on the FMRI index, indicating its significant bioactive potential.

In the case of *Rosa laevigata* Michx., also valued with a 5 on the FMRI index [41], scientific studies have evidenced the presence of 450.1 μg/g gallic acid, 2324.6 μg/g catechin, 182.4 μg/g rutin, 20.8 μg/g quercetin, 8.2 μg/g kaempferol, and 12.1 μg/g apigenin. The main components of this fruit include triterpenoids, flavonoids, and polysaccharides. Notably, the total saponins present in *R. laevigata* have been shown to exhibit significant protective activity against acetaminophen-induced liver disorder, as demonstrated by the downregulation of serum ALT and AST levels [47].

Given that the health benefits of a diet rich in bioactive compounds, especially in protecting against cardiovascular diseases, are largely attributed to the ability of these compounds to mitigate oxidative stress [78,79], it is plausible to suggest that the consumption of certain wild edible fruits rich in phenolic compounds could help reduce the risk of diseases linked to oxidative damage. Expanding the study of the potential of edible wild fruits as a source of phenolic compounds would broaden our understanding of their health benefits. For instance, studies on lesser-known wild species from the Amazon (*Clidemia capitellata* (Bonpl.) D.Don, *Clidemia hirta*, *Clidemia japurensis*, *Tococa bullifera*, *Clidemia pustulata*, and *Clidemia rubra*) have shown antioxidant potential, with total phenolic compound contents ranging from 7.0 to 290.6 mg GAE/100 g fresh weight. Although these results are comparable to the total phenolic content of goji berries (295.7 mg GAE/100 g fresh weight) or raspberries (223.2 mg GAE/100 g fresh weight), they were generally lower compared to more widely studied fruits like *Vaccinium macrocarpon* (cranberry), with 350.5 mg GAE/100 g fresh weight, or *Vaccinium angustifolium* (blueberry), with 371.3 mg GAE/100 g fresh weight [45]. Conversely, nutritional analysis of wild species from the Amazon revealed compositional profiles comparable to more widely studied berries, with similar moisture, protein, and carbohydrate contents. However, the higher ash content observed in these species suggests potentially elevated mineral concentrations, warranting further investigation.

At this juncture, it is pertinent to consider that numerous studies have consistently demonstrated a significantly higher content of secondary metabolites, primarily bioactive compounds, in wild plants relative to their cultivated counterparts [80,81]. This phenomenon is primarily attributable to the exposure of wild plants to adverse environmental conditions, which induces the biosynthesis of these compounds as a defense mechanism. Supporting this assertion, the total phenolic content in wild *Prunus avium* was found to be notably higher than that reported for cultivated sweet cherries [82]. Furthermore, a comparative study of wild and cultivated *Vaccinium* extracts revealed that the wild extracts exhibited a 3.04-fold increase in antioxidant activity, as determined by EC_50_ values [83].

Furthermore, considering the nutritional value of edible wild fruits, it is important to note that while wild food foraging may not be the primary source of sustenance in the modern world, it can play a valuable role in supplementing diets in areas with limited food access [58]. Consequently, the study and consumption of wild edible species can contribute to addressing malnutrition in vulnerable populations, particularly in regions with limited access to diverse and nutrient-dense foods [40].

Additionally, species such as *T. bullifera* have not yet been explored as food sources, presenting a significant opportunity for further investigation and promotion. For instance, *Quercus* spp. (oak acorns) are primarily used as animal feed, but there is increasing interest in their potential as a food source for humans [84]. In addition to their potential as alternatives to other agricultural products, their consumption could also have an economic impact on regions where these wild species are abundant, contributing to local economies and fostering sustainable agricultural practices [46]. This is supported by historical evidence, as observed in past societies where the nutritional value of acorns was recognized as comparable to that of cereals.

In the case of some wild fruits, such as *Clidemia capitellata*, while not showing significant antioxidant activity or high levels of bioactive compounds, their proximate composition revealed that they are sweet, despite their low energy content (65 Kcal/100 g fw) [45]. Carbohydrates were the most prominent macronutrient in these fruits (13.43 g/100 g fw). Although not widely associated with known health benefits, the fruit could serve as a source of natural sweeteners, a potential recognized by other researchers who have identified wild species as possible sources of food additives [85,86]. In summary, the utilization of wild edible fruits contributes to both health promotion and nutritional well-being, as they are considered valuable sources of essential nutrients and bioactive compounds. Their use reaffirms traditional culinary practices and, importantly, promotes environmental sustainability and biocultural conservation [87]. Wild fruits, especially small ones, represent an underutilized and ecologically sound source of nutrients, especially in times of food insecurity, when vulnerable populations are at risk.

The growing interest in the procurement, processing, and consumption of wild foods reflects a wider public trend toward healthier diets. As a result, the scientific community is increasingly focused on evaluating the pharmacological and nutritional properties of these fruits, viewing them as promising alternatives for the production of natural and sustainable food additives [52]. Furthermore, there is a strong emphasis on recognizing and preserving the traditional knowledge and practices associated with these foods [88].

However, despite the growing trend among researchers to promote the revaluation of wild plants due to their potential benefits, it is crucial to acknowledge that some fruits may contain antinutrients or toxic compounds. These substances can include oxalic acid, pyrrolizidine alkaloids, cucurbitacins, saponins, anthracene derivatives, monoterpenes, phenylpropanoids, and prenylflavonoids [89].

For instance, a study on the nutritional, mineral, and vitamin composition of *Eriosema chinense* Vogel revealed the presence of antinutrients such as oxalate, phytate, saponins, and tannins in quantities of 0.18, 0.64, 0.05, and 0.72, respectively [90]. Although these concentrations may not be immediately harmful, their presence can significantly impact the bioavailability of other essential nutrients [91].

Similarly, while the fruits of *Bryonia dioica* Jacq. have been traditionally used as an ointment for treating rheumatic and muscular pain [92], extracts from these fruits have been found to have lethal effects, potentially due to the presence of bridiofin [93].

Consequently, as some researchers have noted, there is a continuing need for comprehensive investigations into the phytochemical content, antioxidant potential, and the presence of both essential and toxic components in conventional food resources. Additionally, nutritional characterization and the assessment of antinutrient content remain crucial areas of research [94,95].

## 6. Conclusions

In this study, a comprehensive review of international treaties addressing sustainable food systems was conducted in response to the growing global demand for sustainable agricultural practices, biodiversity conservation, and climate change mitigation to safeguard food security. A total of 12 international and European treaties or strategies with strong relevance to wild edible fruits were identified. Over the years, regulatory guidelines have increasingly promoted sustainable food systems. As fruits are the most commonly consumed part of wild plants, their revalorization offers a promising approach to achieving the goals of these strategies.

It can be affirmed that a great effort has been made to preserve the biodiversity of wild edible fruits, but it is now necessary to establish new frameworks or reinforce existing ones to ensure a sustainable agri-food system that takes a multidisciplinary perspective (biodiversity, food, and agriculture).

In a scientific literature review on the sustainability and health aspects of wild edible fruits, over 100 references link wild edible fruits to human health, emphasizing their role as sources of bioactive compounds with significant health benefits. With the growing prominence of international sustainability agreements, studies increasingly explore the dual advantages of wild edible fruits for both health and environmental sustainability. However, despite the extensive literature, less than 15% of studies address sustainability aspects. This indicates that most research focuses primarily on the nutritional and functional properties of these fruits, often neglecting their contributions to biodiversity conservation, the preservation of traditional knowledge, and the enhancement of local economies.

The use of wild resources, especially edible wild fruits, dates back to prehistoric times, fostering deep and extensive knowledge of their medicinal and nutritional value. While numerous studies highlight the health benefits of wild fruits, there remains a lack of scientific evidence demonstrating their broader positive impacts on environmental sustainability and biodiversity. Integrating these fruits into sustainable practices could be pivotal for supporting future food security and community well-being.

Harnessing wild plant species, especially knowing the potential of wild fruits, represents a critical strategy for enhancing genetic diversity in modern crops, with the potential to improve food security sustainably. It includes selecting the right wild fruits, adapting them, cultivating them, scaling their growth, marketing them, distributing them, etc. Beyond their health benefits, wild edible fruits offer an opportunity to advance sustainable food systems. These underutilized resources represent a promising nutritional option, particularly in regions facing food insecurity. Incorporating wild fruits into food systems can diversify diets, alleviate pressure on conventional agriculture, and contribute to more resilient and sustainable food production.

Moreover, the incorporation of wild fruits into our food system could bring health benefits due to their rich nutritional content and antioxidant bioactive compounds that enhance bodily functions. The characterization of wild edible fruits could also help identify the presence of antinutritional or toxic compounds in certain species.

While wild fruits may offer significant benefits, it is crucial to balance traditional knowledge with scientific research to ensure the conservation of these species. Special attention should be paid to potential threats, particularly over-harvesting. The establishment of international agreements that address threats such as agricultural expansion, timber extraction, overexploitation, overgrazing, and the impact of invasive species is essential for ensuring the sustainable use of wild edible fruits.

## Figures and Tables

**Table 1:** Literature PubMed search results on the sustainability and health aspects of wild edible fruits.

Keywords	Number of References
“Human health” and “sustainability”	127,912
“Wild edible fruits” and “human health”	119
“Sustainability” and “wild edible fruits”	91
“Sustainability”, “wild edible fruits”, and “human health”	18

**Table 2:** Overview of the sustainability aspects of various wild edible fruits organized according to the proposed criteria.

Wild Edible Fruit	Sustainability Pillars			Reference
	EN	S	EC	
*Alibertia patinoi* (Cuatrec.) Delprete & C.H.Perss, *Bactris gasipaes* Kunth, *Eugenia stipitata* McVaugh, *Pourouma cecropiifolia* Mart, *Solanum sessiliflorum* Dunal	X	X	X	[38]
*Balanites aegyptiaca* (L.) Delile, *Carissa spinarum* L., *Ximenia americana* L.	X	X		[39]
*Berberis angulosa* Wall., *Berberis aristata* DC, *Berberis ceratophylla* G. Don, *Fragaria nubicola* Lindl. *ex Lacaita*, *Hippophae tibetana* Schltdl., *Ribes orientale* Desf., *Zanthoxylum armatum* DC.	X	X	X	[40]
*Camellia drupifera* Lour., *Camellia euphlebia* Merr. *ex Sealy*, *Camellia indochinensis var. tunghin ensis* (Hung T.Chang) T.L. Ming & W.J. Zhang, *Camellia oleifera* C. Abel, *Camellia petelotii* (Merr.) Sealy, *Campanumoea javanica* Bl., *Canarium pimela* K.D.Koeni, *Castanopsis hystrix* Miq., *Choerospondias axillaris* (Roxb.) B. L. Burtt & A. W. Hill, *Citrus limonia* Osb, *Cyclocodon lancifolius* Kurz., *Embelia laeta* (L.) Mez, *Embelia ribes* Burm.f, *Embelia ribes* subsp. *pachyphylla* (Chun ex C. Y. Wu & C. Chen) Pipoly & C. Chen, *Ficus auriculata* Lour, *Ficus oligodon* Miq, *Ficus pumila* L., *Garcinia multiflora* Champ. ex Benth, *Garcinia oblongifolia* Champ. ex Benth, *Gardenia jasminoides* J.Ellis, *Glycosmis pentaphylla* (Retz.) DC, *Ligustrum lucidum* W.T.Aiton, *Litsea cubeba* Pers., *Lithocarpus pachylepis* A. Camus, *Macrosolen cochinchinensis* Blume, *Melastoma malabathricum* L., *Melastoma sanguineum* Sims., *Momordica subangulata* Blume, *Osyris lanceolata* Hochst. & Steud, *Psidium guajava* L., *Pyrus calleryana* Decne., *Rosa laevigata* Michx., *Rubus alceifolius* Poiret in Lamarck, *Rubus cochinchinensis* Tratt., *Rubus leucanthus* Hance., *Rubus pluribracteatus* L. T. Lu & Boufford, *Rubus rosifolius* Sm, *Sageretia thea* (Osbeck) M.C.Johnst, *Saurauia tristyla* DC., *Ulmus pumila* L.	X	X	X	[41]
*Capparis tomentosa* Lam., *Carissa spinarum* L., *Cordia africana* Lam., *Embelia schimperi* Vatke., *Ficus sur* Forssk., *Ficus sycomorus* L., *Flueggea virosa* (Roxb. ex Willd.) Royle, *Gardenia ternifolia* Schu mach. & Thonn., *Justicia schimperiana* (Hochst. ex Nees) T. Anderson., *Momordica foetida* Schumach., *Nauclea latifolia* Sm., *Rubus lambertianus Ser*, *Rumex nervosus* Vahl., *Senna petersiana* (Bolle) Lock., *Solanum villosum* Mill., *Ximenia americana* L.	X	X	X	[42]
*Castanea mollissima* Blume, *Cucumis sativus* L., *Cucumis sativus* L., *Eriobotrya japonica* (Thunb.) Lindl., *Ficus tikoua* Bur., *Juglans regia* L., *Kadsura longipedunculata* Finet & Gagnepain, *Ligustrum lucidum* W.T.Aiton, *Litsea cubeba* Pers, *Luffa aegyptiaca* Miller, *Maclura cochinchinensis* (Lour.) Corner, *Melastoma dodecandrum* Lour., *Melastoma malabathricum* L., *Myrica rubra* Siebold & Zuccarini, *Prunus persica* L., *Pyracantha fortuneana* (Maximowicz) H. L. Li, *Rubus alceifolius* Poiret in Lamarck, *Rubus niveus* Thunb., *Rubus parvifolius* L., *Rubus rosifolius* Stokes, *Solanum lycopersicum* L., *Vitis balansana* Planchon., *Zanthoxylum armatum* DC.	X	X	X	[43]
*Phyllanthus emblica* L., *Piper longum L.*, *Semecarpus anacardium* L.f., *Terminalia bellirica* (Gaertn.) Roxb.	X	X	X	[44]
*Clidemia hirta* (L.) D. Don, *Clidemia japurensis* DC, *Clidemia pustulata* DC, *Clidemia rubra* (Aubl.) G.Don f, *Tococa bullifera* Mart. & Schrank ex DC.	X	X	X	[45]
*Quercus* spp.	X	X	X	[46]
*Rosa laevigata* Michx.	X	X	X	[47]

Pillars of sustainability: environmental (EN), social (S), and economic (EC) dimensions. Proposed criteria compliance (X).

**Table 3:** Review of the literature on health aspects of wild edible fruits.

Plant Food	Health Aspect	Reference
Health aspects of wild edible fruits’ use in traditional medicine		
*Alibertia patinoi* (Cuatrec.) Delprete & C.H.Perss.	These fruits are marketed as a ‘superfood’ with purported energizing and aphrodisiac properties.	[38]
*Balanites aegyptiaca* L. Delile	The fruits are regarded as a nutraceutical component of a wild edible plant traditionally used to treat epistaxis (nosebleeds), headaches, and stomach-aches.	[39]
*Berberis angulosa* Wall.	These fruits are known to be rich in vitamins.	[40]
*Berberis aristate* DC	These fruits are known to be rich in vitamins.	[40]
*Berberis ceratophylla* G. Don	These fruits are known to be rich in vitamins.	[40]
*Camellia drupifera* Lour	The Food–Medicinal Role Index of these fruits is valued at 4.	[41]
*Camellia euphlebia* Merr. ex Sealy	The Food–Medicinal Role Index of these fruits is valued at 3.	[41]
*Camellia indochinensis var. tunghin ensis* (Hung T.Chang) T.L.Ming & W.J.Zhang	The Food–Medicinal Role Index of these fruits is valued at 3.	[41]
*Camellia oleifera* C.Abel	In the CFSI, the Food–Medicinal Role Index value for extracts containing oil is 4.	[41]
*Camellia petelotii* (Merr.) Sealy	In the CFSI, the Food–Medicinal Role Index value for oil extracts from this plant is 3.	[41]
*Campanumoea javanica* Bl.	The fruit can be consumed directly, with a Food–Medicinal Role Index of 3 in the CFSI.	[41]
*Canarium pimela* K.D.Koenig	The fruit can be marinated, and in the CFSI formula, its Food–Medicinal Role Index remains 3.	[41]
*Capparis tomentosa* Lam.	This plant is considered a nutraceutical.	[42]
*Carissa spinarum* L.	The fruits are regarded as a nutraceutical wild edible used in the treatment of amoebiasis and inflammation. Unripe and roasted fruits, including seeds, were traditionally swallowed to expel *Ascaris*.	[39,42]
*Castanea mollissima* Blume	The Food–Medicinal Role Index of these fruits is valued at 3.	[43]
*Castanopsis hystrix* Miq.	The Food–Medicinal Role Index of these fruits is valued at 3.	[41]
*Choerospondias axillaris* (Roxb.) B. L. Burtt & A. W. Hill	The fruit can be consumed directly, with an FMRI of 5 in the CFSI formula.	[41]
*Citrus limonia* Osb	The fruit can be consumed directly, with an FMRI of 5 in the CFSI formula.	[41]
*Cordia africana* Lam.	This plant is considered a nutraceutical. Traditionally, unripe and roasted fruits, including seeds, were swallowed to expel *Ascaris*.	[42]
*Cucumis sativus* L.	The Food–Medicinal Role Index of these fruits is valued at 4.	[43]
*Cyclocodon lancifolius* Kurz	The Food–Medicinal Role Index of these fruits is valued at 3.	[41]
*Embelia laeta* (L.) Mez	The Food–Medicinal Role Index of these fruits is valued at 3.	[41]
*Embelia ribes* Burm.	The Food–Medicinal Role Index of these fruits is valued at 3.	[41]
*Embelia ribes* subsp. *pachyphylla* (Chun ex C. Y. Wu & C. Chen) Pipoly & C. Chen	The Food–Medicinal Role Index of these fruits is valued at 3.	[41]
*Embelia schimperi* Vatke.	Regarded as a nutraceutical plant, its fruits, including seeds, were traditionally used to treat tapeworm infestation.	[42]
*Eriobotrya japonica* (Thunb.) Lindl.	In CFSI, the value of the Food–Medicinal Role Index is 5.	[43]
*Ficus auriculata* Lour.	The Food–Medicinal Role Index of these fruits is valued at 3.	[41]
*Ficus oligodon* Miq	The Food–Medicinal Role Index of these fruits is valued at 3.	[41]
*Ficus pumila* L.	The Food–Medicinal Role Index of these fruits is valued at 3.	[41]
*Ficus sur* Forssk.	This is considered to be a nutraceutical plant.	[42]
*Ficus sycomorus* L.	This is considered to be a nutraceutical plant.	[42]
*Ficus tikoua* Bur.	The Food–Medicinal Role Index of these fruits is valued at 3.	[43]
*Flueggea virosa* (Roxb. ex Willd.) Royle	This plant is considered a nutraceutical.	[42]
*Fragaria nubicola* Lindl. ex Lacaita	It is traditionally well known among the residents of Manang for being highly nutritious.	[40]
*Garcinia mul tiflora* Champ. ex Benth	The Food–Medicinal Role Index of these fruits is valued at 3.	[41]
*Garcinia oblongifolia* Champ. ex Benth	The Food–Medicinal Role Index of these fruits is valued at 3.	[41]
*Gardenia jasminoides* J.Ellis	The Food–Medicinal Role Index of these fruits is valued at 5.	[41]
*Gardenia ternifolia* Schu mach. & Thonn.	This plant is considered a nutraceutical.	[42]
*Glycosmis pentaphylla* (Retz.) DC	The Food–Medicinal Role Index of these fruits is valued at 3.	[41]
*Hippophae tibetana* Schltdl.	The residents of Manang (Nepal) are aware that the fruit can be used to produce a healthy, vitamin-rich juice.	[40]
*Hippophae salicifolia* D.Don	The residents of Manang (Nepal) are aware that the fruit can be used to produce a healthy, vitamin-rich juice.	[40]
*Juglans regia* L.	The Food–Medicinal Role Index of these fruits is valued at 3.	[43]
*Justicia schimperiana* (Hochst. ex Nees) T. Anderson.	This plant is considered a nutraceutical.	[42]
*Kadsura longipedunculata* Finet & Gagnepain	The Food–Medicinal Role Index of these fruits is valued at 4.	[43]
*Ligustrum lucidum* W.T.Aiton	The Food–Medicinal Role Index of these fruits is valued at 5.	[41,43]
*Lithocarpus pachylepis* A. Camus	The Food–Medicinal Role Index of these fruits is valued at 3.	[41]
*Litsea cubeba* Pers.	The Food–Medicinal Role Index of these fruits is valued at 5.	[41,43]
*Luffa aegyptiaca* Miller	The Food–Medicinal Role Index of these fruits is valued at 3.	[43]
*Maclura cochinchinensis* (Lour.) Corner	The Food–Medicinal Role Index of these fruits is valued at 4.	[43]
*Macrosolen cochinchinensis* (Lour.) Tiegh	The Food–Medicinal Role Index of these fruits is valued at 5.	[41]
*Melastoma dodecandrum* Lour.	The Food–Medicinal Role Index of these fruits is valued at 3.	[43]
*Melastoma malabathricum* L.	The Food–Medicinal Role Index of these fruits is valued at 3.	[41,43]
*Melastoma sanguineum* Sims.	The Food–Medicinal Role Index of these fruits is valued at 3.	[41]
*Momordica foetida* Schumach.	This plant is considered a nutraceutical.	[42]
*Momordica subangulata* Blume	The Food–Medicinal Role Index of these fruits is valued at 4.	[41]
*Myrica rubra* Siebold & Zuccarini	The Food–Medicinal Role Index of these fruits is valued at 3.	[43]
*Nauclea latifolia* Sm.	This plant is considered a nutraceutical.	[42]
*Osyris lanceolata* Hochst. & Steud	The Food–Medicinal Role Index of these fruits is valued at 3.	[41]
*Phyllanthus emblica* L.	Fruits are used to treat cough and cold.	[44]
*Piper longum* L.	Fruit powder is used for the treatment of cough and cold.	[44]
*Prunus persica* L.	The Food–Medicinal Role Index of these fruits is valued at 3.	[43]
*Psidium guajava* L.	The Food–Medicinal Role Index of these fruits is valued at 3.	[41]
*Pyracantha fortuneana* (Maxim.) H. L. Li	The Food–Medicinal Role Index of these fruits is valued at 3.	[43]
*Pyrus calleryana* Decne.	The Food–Medicinal Role Index of these fruits is valued at 4.	[41]
*Rosa laevigata* Michx.	The Food–Medicinal Role Index of these fruits is valued at 5.	[41]
*Ribes orientale* Desf.	The fruits are rich in vitamins.	[40]
*Rubus alceifolius* Poir.	The Food–Medicinal Role Index of these fruits is valued at 3.	[41,43]
*Rubus cochinchinensis* Tratt.	The Food–Medicinal Role Index of these fruits is valued at 3.	[41]
*Rubus lambertianus* Ser.	The Food–Medicinal Role Index of these fruits is valued at 3.	[43]
*Rubus leucanthus* Hance.	The Food–Medicinal Role Index of these fruits is valued at 3.	[41]
*Rubus niveus* Thunb.	The Food–Medicinal Role Index of these fruits is valued at 3.	[43]
*Rubus parvifolius* L.	The Food–Medicinal Role Index of these fruits is valued at 3.	[43]
*Rubus pluribracteatus* L. T. Lu & Boufford	The Food–Medicinal Role Index of these fruits is valued at 3.	[41]
*Rubus rosifolius* SM	The Food–Medicinal Role Index of these fruits is valued at 3.	[41,43]
*Rumex nervosus* Vahl.	This plant is considered a nutraceutical.	[42]
*Sageretia thea (Osbeck)* M.C.Johnst	The Food–Medicinal Role Index of these fruits is valued at 3.	[41]
*Saurauia tristyla* DC.	The Food–Medicinal Role Index of these fruits is valued at 3.	[41]
*Semecarpus anacardium* L.f.	The seeds are used to treat cuts and wounds.	[44]
*Senna petersiana* (Bolle) Lock.	This plant is considered a nutraceutical.	[42]
*Solanum lycopersicum* L.	The Food–Medicinal Role Index of these fruits is valued at 3.	[43]
*Solanum lyratum* Thunb.	The Food–Medicinal Role Index of these fruits is valued at 3.	[43]
*Solanum villosum* Mill.	This plant is considered a nutraceutical.	[42]
*Terminalia bellirica* (Gaertn.) Roxb	Fruit powder is used for the treatment of cough.	[44]
*Ulmus pumila* L.	The Food–Medicinal Role Index of these fruits is valued at 3.	[41]
*Vitis balansana* Planch.	The Food–Medicinal Role Index of these fruits is valued at 5. Local inhabitants believe that these beverages promote blood circulation, stimulate metabolism, and have a beneficial effect on overall health.	[41]
*Ximenia americana* L.	The fruits are regarded as a nutraceutical wild edible, traditionally used to treat tooth diseases and abdominal pain.	[39,42]
*Zanthoxylum armatum* DC.	Residents of Manang (Nepal) possess extensive knowledge of traditional medicinal practices used to treat a variety of ailments, including colds, dysentery, and altitude sickness. The Food–Medicinal Role Index of these fruits is valued at 5.	[40,43]
Health aspects of wild edible fruits by scientific evidence		
*Alibertia patinoi* (Cuatrec.) Delprete & C.H.Perss	They demonstrate antimicrobial, antitumor, cytotoxic, spermicidal, and skin-protective activities.	[38]
*Bactris gasipaes* Kunth	The fruits of *Bactris gasipaes* exhibit anti-inflammatory, antimicrobial, and hepatoprotective activities.	[38]
*Camellia oleifera* C.Abel	*Camellia oleifera* has a high concentration of unsaturated fatty acids, which are associated with the prevention and treatment of cardiovascular and cerebrovascular diseases.	[50]
*Clidemia hirta* (L.) D.Don	Studies have demonstrated the fruit’s high total phenolic content and significant antioxidant activity across various assays.	[45]
*Clidemia japurensis* DC	Studies have demonstrated the fruit’s high total phenolic content and significant antioxidant activity across various assays.	[45]
*Clidemia pustulata* DC	Studies have demonstrated the fruit’s high total phenolic content and significant antioxidant activity across various assays.	[45]
*Clidemia rubra* (Aubl.) G.Don f,	A study has demonstrated their high total phenolic content.	[45]
*Eugenia stipitate* McVaugh	Extracts and powdered fruits are associated with antigenotoxic and antimutagenic activities. Anthocyanin-rich extracts demonstrate activity against laryngeal, liver, and breast cancer cell lines. Additionally, hydroalcoholic extracts inhibit acetylcholinesterase, which is of interest for improving symptoms of Alzheimer’s disease.	[38]
*Pourouma cecropiifolia* Mart	Studies on the biological activity of extracts and powders show that they exhibit cytotoxic activity.	[38]
*Quercus* spp.	A review highlights the beneficial effects on human health attributed to the biological activities of phenolic compound extracts, including antiseptic, antimicrobial, antioxidant, antiproliferative, apoptosis-inducing, anti-inflammatory, antitumoral, gastroprotective, antiviral, cytotoxic, genotoxic, hemolytic, anti-hemorrhoidal, neuroprotective, anti-cholinesterase, and hepatoprotective activities.	[46]
*Rosa laevigata* Michx.	Fresh fruits of *Rosa laevigata* are rich in vitamin C and contain eight essential amino acids necessary for the human body.	[47]
*Rumex nervosus* Vahl.	This plant is considered a nutraceutical, with potential as a wild edible plant that provides a rich source of phenolics, flavonoids, and antioxidant compounds, making it useful for treating oxidative stress-related diseases.	[51]
*Solanum sessiliflorum* Dunal	Extracts and powdered fruits exhibit in vitro biological activity against lipid peroxidation, which is of particular interest in relation to metabolic syndrome.	[38]
*Tococa bullifera* Mart. & Schrank ex DC	A study has demonstrated their high total phenolic content.	[45]

Cultural Significance of Wild Edibles (CFSI) index, used to evaluate the potential of wild edible resources. The index incorporates the Food–Medicinal Role Index (FMRI), which classifies wild edibles based on their health benefits and medicinal properties. The FMRI categories include the following: ‘very high or medicinal food’ (value 5), ‘high or medicine for treating a specific disease’ (value 4), ‘moderately high or very healthy food’ (value 3), ‘moderately low, healthy food or unknown efficacy’ (value 2), and ‘unknown or possibly toxic’ (value 1).

## Abbreviations

The following abbreviations are used in this manuscript:

FAO	Food and Agriculture Organization of the United Nations
CBD	Convention on Biological Diversity
WHO	World Health Organization
SDGs	Sustainable Development Goals
CITES	Convention on International Trade in Endangered Species of Wild Fauna and Flora
UNEP	United Nations Environment Programme
TRIPS	Agreement on Trade-related Aspects of Intellectual Property Rights
WTO	World Trade Organization
MDGs	Millennium Development Goals
IUCN	International Union for Conservation of Nature
UNEP-WCMC	United Nations Environment Programme World Conservation Monitoring Centre
IT-PGRFA	International Treaty on Plant Genetic Resources for Food and Agriculture
COP	Conference of the Parties
ABS	Access to Genetic Resources and Fair and Equitable Sharing of Benefits Arising from Their Utilization
BFA	Biodiversity for food and agriculture
CFSI	Cultural Significance of Wild Edibles
FMRI	Food–Medicinal Role Index
SPs	Silvopastoral systems
